# Size, more than oxygen need, determines the number of capillaries around a muscle fibre

**DOI:** 10.1113/EP092911

**Published:** 2025-06-08

**Authors:** Christopher S. Fry, Carlo Reggiani

**Affiliations:** ^1^ Center for Muscle Biology University of Kentucky Lexington Kentucky USA; ^2^ Department of Biomedical Sciences University of Padova Padova Italy; ^3^ ZRS, Science and Research Center, Garibaldijeva 1, 6000 Koper Slovenia

**Keywords:** capillary vessels, microcirculation, skeletal muscle

1

In the present issue of *Experimental Physiology*, the paper by Degens et al. (2026) provides important insight into our understanding of the structure–function relationship of the capillary network around skeletal muscle fibres and contributes to a scientific debate that has spanned more than a century (Clark et al., 2008; Poole et al., 2008).

Although all organs, from brain to liver, change their metabolism in close relationship with functional activity, skeletal muscle is remarkable because the transition from rest to full contractile activity might require an increase of ATP regeneration rate of >100‐fold. This would imply a proportional increase in muscle blood flow to facilitate gaseous exchange, lactate clearance and nutrient delivery to the contracting fibres (Andersen & Saltin, 1985). There is no doubt that, at the organ level, this increase in muscle blood flow is facilitated by strong arteriolar vasodilatation and a reduction in resistance, but less clear is the counterpart at the level of the microcirculation.

The most widespread models of control of the microcirculation over the past century were based upon the Nobel prizewinning August Krogh's ‘capillary motor’ hypothesis (Krogh, 1919). Krogh's work postulated that most capillaries along muscle fibres are closed at rest but open to enhance red blood cell flux during contraction. Precapillary sphincters, termed Rouget cells (now known as pericytes), are responsible for opening and closing the access to capillaries.

However, the last two decades have witnessed a large change in our understanding of the function of the microcirculation in skeletal muscle that directly challenges the capillary recruitment theory (Poole & Musch, 2023). Within the context of oxygen supply, an increase in oxygen extraction from haemoglobin from 25% to 90% occurs during contractile activity in the presence of accelerated blood flow velocity. This might appear counterintuitive, because a higher flow velocity reduces the transit time but at the same time increases intracapillary haematocrit and haemoglobin oxygenation at the venous end. In this way, oxygen extraction is facilitated along the length of the capillary with more red blood cells present within the capillary. Moreover, the transport from red blood cells to the interstitial compartment is enhanced as the oxygen partial pressure gradient is increased, and red blood cell membrane crossing is facilitated via aquaporins in addition to simple diffusion.

Recent advancements in imaging have revealed that mitochondria exist within an interconnected reticulum extending from the subsarcolemmal region to the most capillary‐distant inter‐myofibrillar mitochondria within skeletal muscle fibres (Parry et al., 2024). The highest concentration of subsarcolemmal mitochondria is closest to capillaries, which are enriched in Complex IV, whereas Complex V is more abundant in the inter‐myofibrillar mitochondria.

In the evolving framework of the skeletal muscle microcirculation, the number and the location of capillaries around individual fibres become relevant in relationship to the myofibre size and oxygen requirement for aerobic metabolism. It is likely that there are precise constraints to the correct interaction between those three factors.

Degens et al. (2026) explore capillary density around a myofibre, their relationship to the size of the myofibre, and their impact on its aerobic metabolism, using muscle samples from mice (thin fibres from diaphragm, soleus and extensor digitorum longus) and humans (larger fibres from vastus lateralis and soleus). Human samples included samples from resistance‐trained subjects with fibre cross‐sectional area of >25 000 µm^2^ and endurance‐trained subjects with greater mitochondrial abundance and an elevated capillary network. In comparison to human muscle fibres, the murine fibres are not only thinner but also have higher aerobic metabolic activity, assessed by succinate dehydrogenase (SDH) activity. The dataset encompasses an exceptional range in muscle fibre size and mitochondrial abundance.

An area‐based approach, with measurement of Voronoi polygons (equivalently known as capillary domains), was adopted to avoid the limitations of the morphometric analysis based on quantification of capillaries and myofibres on whole microscopic sections and yielding parameters such as mean capillary density per millimetre squared or mean number of capillaries per fibre. A capillary domain was defined as the area of a muscle cross‐section surrounding an individual capillary and closer to that capillary than to all neighbouring capillaries.

The number of capillaries around a fibre (CAF) was found to increase in relationship to fibre cross‐sectional area, thus the capillary fibre density [CFD, at fibre level; CFD = CAF/cross‐sectional area (CSA)] decreases with increased CSA. These observations hold true irrespective to animal species, fibre type and level of oxidative metabolism.

The local capillary‐to‐fibre ratio is the sum of the capillary domain fractions contributing to a given fibre, which also increases in proportion to fibre CSA, with the slope being steeper in murine than in human fibres. However, the murine and human slopes become equal if local capillary‐to‐fibre ratio is plotted against the total mitochondrial activity (OD‐INT), which is obtained as the product of optical density of SDH staining (OD) by fibre CSA. This shows that local capillary‐to‐fibre ratio is proportional to the total oxidative activity regardless of species and fibre types. The independence of fibre type, although outwardly surprising, is in line with prior work (Ahmed et al., 1997), finding that fibre type has little, if any, impact on the capillary supply to a fibre.

Finally, when optical density of SDH staining is plotted against fibre CSA, a hyperbolic relationship appears over the whole range of fibre size. This confirms the size principle, which states that aerobic metabolic activity, as estimated by SDH activity, is associated with small fibre size (Van der Laarse et al., 1998). Importantly, this is not inconsistent with the observations that small and intensely aerobic mouse fibres are surrounded by fewer capillaries than large human fibres.

Degens et al. (2026) address a long‐standing question: Can increasing capillary density and oxidative capacity overcome the diffusion limitations associated with larger muscle fibres? The results of the work demonstrate that there are physiological and geometric upper limits to how much oxidative capacity and capillary supply can scale with fibre cross‐sectional area. The constraints identified can broadly be classified as diffusion and structural limitations. Within diffusion constraints, oxygen and/or substrate diffusion cannot be maintained beyond a certain fibre size, regardless of mitochondrial density. For structural constraints, physical capillary placement around fibres cannot reduce diffusion distances beyond a certain point.

## AUTHOR CONTRIBUTIONS

Carlo Reggiani and Christopher S. Fry contributed to the conception of the Viewpoint. Each author participated in designing, writing and revising the Viewpoint, approved the final version and agrees to be accountable for all aspects of the work in ensuring that questions related to the accuracy or integrity of any part of the work are appropriately investigated and resolved. All persons designated as authors qualify for authorship, and all those who qualify for authorship are listed.

## CONFLICT OF INTEREST

The authors declare no conflicts of interest.

## FUNDING INFORMATION

None.
